# Fear of future workplace violence and its influencing factors among nurses in Shandong, China: a cross-sectional study

**DOI:** 10.1186/s12912-021-00644-w

**Published:** 2021-07-07

**Authors:** Chang Fu, Yaru Ren, Guowen Wang, Xiuxin Shi, Fenglin Cao

**Affiliations:** 1 Department of Health Psychology, School of Nursing and Rehabilitation, Cheeloo College of Medicine, Shandong University, No. 44 Wenhuaxilu Rd., Jinan, 250012 Shandong China; 2 Department of Nursing Science, Shandong Provincial Qianfoshan Hospital Affiliated to Shandong First Medical University, No. 16766 Jingshilu Rd., Jinan, 250014 Shandong China; 3 Department of Education, Shandong Provincial Hospital Affiliated to Shandong First Medical University, Jinan, No. 324 Jingwuweiqilu Rd., Jinan, 250021 Shandong China; 4 Office of Medical Quality Control, Qilu Hospital, Cheeloo College of Medicine, Shandong University, No. 107 Wenhuaxilu Rd., Jinan, 250012 Shandong China

**Keywords:** Nurse, Workplace violence, Fear of future workplace violence, Influencing factors

## Abstract

**Background:**

Fear of workplace violence has become a critical issue worldwide, which can lead to burnout, low levels of job satisfaction, and turnover. However, to date, little attention has been directed toward fear of workplace violence among nurses. Accordingly, this study investigated the level of fear of future workplace violence and its influencing factors among nurses in Shandong, China.

**Methods:**

A cross-sectional study was conducted from July 30 through September 30, 2020 in Shandong Province, China. A total of 1898 nurses were enrolled from 12 tertiary hospitals. Fear of future workplace violence was measured using the Fear of Future Violence at Work scale. Demographic information, employment characteristics, social support, and experience of workplace violence were assessed. Multiple linear regression analysis was used to explore the influencing factors of fear of future workplace violence.

**Results:**

The average score of fear of future violence at work was 67.43 ± 17.20 among nurses. Multiple linear regression analysis showed that higher fear of future violence at work scores were reported among nurses who were female (B = 7.10, *p* < 0.001), married (B = 3.50, *p* = 0.028), with a monthly income ≥5000 Chinese yuan (CNY) (B = 3.14, *p* = 0.007), working in the department of internal medicine (B = 2.90, *p* = 0.032), surgery (B = 5.03, *p* < 0.001), pediatrics (B = 5.38, *p* = 0.003), or emergency department (B = 4.50, *p* = 0.010), working as a contract employee (B = 2.41, *p* = 0.042), or who had experienced workplace violence (B = 7.02, *p* < 0.001). Lower fear of future violence at work scores were found among nurses who took vacations (1–14 days: B = − 2.52, *p* = 0.047; ≥15 days: B = − 3.69, *p* = 0.007) and had a high-level of social support (B = − 2.03, *p* = 0.020).

**Conclusions:**

There was a high level of fear of future workplace violence among nurses in Shandong, China. This should be considered an important issue by hospital administrators and government officials. Effective interventions need to be enacted to address the influencing factors of fear of future workplace violence.

## Background

Workplace violence (WPV) has been recognized as a global public health issue, especially in health sectors, and has accordingly attracted the attention of researchers worldwide [1]. Nurses are more likely to encounter WPV compared to other healthcare providers [2]. In recent years, the prevalence of WPV among nurses has been increasing [3]. Previous studies reported that the prevalence of WPV among nurses was 43% in the United States [4], 44% in Japan [5], and 67% in Italy [6]. The high prevalence of WPV could lead to an insecure work environment and fear of future WPV among nurses [7].

Fear of future WPV is an emotional response to the individual risk of WPV victimization [8], which has a negative impact on individuals’ health and organizational development. At the individual level, a previous research on trauma and mental health found that a long-lasting feeling of insecurity affects individuals’ physical and psychological well-being [8, 9]. At the organizational level, fear of future WPV is a major source of job stress which can reduce employee productivity [10]. Because of the high prevalence of WPV involving nurses, most of them are worried about the security in their workplace; such a situation will likely distract nurses from their work. In addition, fear of future WPV could increase turnover intentions and hinder the recruitment of medical workers. Due to the fear of violent incidents, a relatively large proportion of nurses’ children choose professions other than healthcare professions [11].

Researchers have focused predominately on the health outcomes and work performance of individuals who experienced WPV [3, 12–14]. However, beyond the direct effects of WPV, an unsafe work environment for medical workers could also lead to heightened insecurity in nurses’ daily lives and could cause them to feel fear of future WPV, including those with no history of experiencing WPV. With the rapid development of virtual internet technology, the speed and ability to obtain information from others have advanced greatly. Incidences of patient-initiated WPV have been repeatedly and widely reported by the public media [15]. In addition to nurses who have been exposed to violence, those who learn the news about serious medical worker-patient conflict may also develop a fear of future WPV. The high prevalence of WPV affects healthcare professionals, not only because of the violence they have experienced but also because of fear of future WPV [16]. Safety should be regarded as a basic human right, which permits individuals to recognize their full potential. In recent years, researchers have investigated fear of future WPV in several professions, such as among social work students [17] and frontline staff from job centers and social security offices [18]. However, little attention has been paid to fear of future WPV and its influencing factors among nurses.

In the past few years, the prevalence of WPV among medical staff has considerably increased and become a social concern in China [19]. Previous studies reported that nearly 70% of Chinese nurses were exposed to WPV [20]. The prevalence was higher than that of developed countries. In China, the stigmatization of medical staff and misleading reports on medical disputes may encourage the prevalence of WPV [3, 21]. Due to the high prevalence of WPV on nurses in China, nurses might feel a relatively high level of fear of future WPV. To maintain the productivity of nurses and improve the quality of the health service they provide, it is important to determine the level of fear of future WPV and its influencing factors among nurses; this was the purpose of the present study, considering nurses in Shandong, China.

## Methods

### Study design, sample, and procedures

The present cross-sectional survey was carried out from July 30 to September 30, 2020 in Shandong, China. Shandong Province is located in East China, with a geographical area of 157,900 km^2^. There are 16 cities at prefecture-level. The population in Shandong Province reached 100.70 million and its gross domestic product (GDP) exceeded one trillion US dollars (USD) by the end of 2019, according to Shandong Provincial Bureau of Statistics [22]. Shandong Province is one of the typical provinces in China in terms of its population structure, social, and culture aspects [23]. A multistage random sampling method was applied for this survey [24]. First, 16 perfecture-level cities were classified into three groups (high, medium, and low) according to per capita GDP level. Two cities were randomly chosen from each group. Second, two tertiary hospitals were randomly chosen in each city. Third, two-thirds of departments, for example, departments of internal medicine, surgery, obstetrics and gynecology, pediatrics, and emergency department, were chosen from each sample hospital [25]. All nurses in the selected departments were invited to join in the survey. The inclusion criteria were voluntary participation by registered nurses who were employed by the hospital. Exclusion criteria were those who were on vacation or absent due to joining in their continuing education, or suffering serious mental or physical disorders that may have hindered their participation.

The minimum sample size was calculated applying a single population proportion formula, *n* = Z_α/2_^2^p(1-p)/d^2^. Based on the assumption: the prevalence of fear of future WPV was 81.9% [26], marginal error of 5, 95% confidence interval (CI) (α = 0.05), *n* = 1.96^2^ × 0.819(1–0.819)/0.05^2^ = 227.79 ≈ 228. In the present study, considering a design effect of 2 (multistage sampling technique) [27] and adding a non-response rate of 20%, the final sample size was 548 nurses [228 × 2 × (1 + 0.2)].

The questionnaire was created applying “*Wenjuanwang*” (*https://www.wenjuan.com/*), a platform of the electronic questionnaires. We sent a web page of the questionnaire to participants’ mobile phones using *WeChat* (a social media app) [24]. The anonymity of participants and the confidentiality of their responses were ensured in the survey. A total of 1933 nurses participated in the present survey. After excluding questionnaires with missing data, 1898 nurses were included for analysis. The effective response rate in the current study was 98.19%.

### Instrument

#### General information

Information about demographic information consisted of age, sex, marital status (married or single/divorced/widowed/separated), educational background (college diploma or below, bachelor’s degree, master’s degree or higher), monthly income, and number of child/children (0, 1, ≥2). Additionally, employment characteristics were investigated, consisting of different departments (e.g. internal medicine, surgery, obstetrics and gynecology, pediatrics, or emergency department), professional title (primary, intermediate, or senior), employment status (regular or contract employee), and vacation time (days per year). Experience of WPV was evaluated by the question, “Have you ever experienced verbal, psychological, or physical violence from patients and/or their relatives, your colleagues and/or supervisors in the hospital?”. Verbal violence included abuse, sarcasm, indignity, effrontery, roar, and so on. Psychological violence included baseless charges or complaints, slander, attempts to damage reputation, destruction of public facilities, booing, maliciously taking pictures, oral or written threats, glowering, waving a clenched fist, stalking, sexual harassment, and so on. Physical violence included biting, pushing, fighting, cutting, sexual assaults, and so on [28–30]. According to the experience of WPV, the nurses were divided into two groups: (1) who had experienced at least one incidence of WPV, and (2) who had not experienced WPV [31].

#### Social support rating scale

Social support was measured applying the Chinese version of the Social Support Rating Scale (SSRS), which has been validated in the Chinese population [32]. It is a 10-item scale comprising three dimensions, i.e., objective support, subjective support, and utilization of support [24]. Questions related to objective support are as follows: “What is your living status in the last year?” (Q2), “What are the sources of financial support and solving problems when you are in an emergency?” (Q6), and “What are the sources of taking comfort and receiving concern when you are in an emergency?” (Q7). Questions on subjective support are as follows: “How many close friends do you have and how much assistance they can provide for you if needed?” (Q1), “How about the relationship among you and your neighbors?” (Q3), “How about the relationship among you and your colleagues?” (Q4), and “How much family support and concern have you received from your family members?” (Q5). Questions on utilization of support are as follows: “What is the way do you talk about trouble?” (Q8), “What is the way to seek assistance from others when you are in trouble?” (Q9), and “How often do you join in activities sponsored by groups (e.g. party organizations, religious organizations, labor unions)?” (Q10). For Q1 ~ Q4 and Q8 ~ Q10, the respondent is asked to select only one choice for each item, scoring 1, 2, 3, or 4 points for choices A, B, C, and D, respectively. Q5 is divided into five subitems (spouse or lover, parents, children, siblings, and other family members). For each subitem, the response “none” scores 1 point, “rare” 2, “general” 3, and “full support” 4. For Q6 and Q7, the answer “no source” scores zero points; otherwise, each source listed scores 1 point [24]. The total scores of SSRS are the sum of the scores of the three subscales. The Cronbach’s alpha coefficient for the SSRS scores in the present survey was 0.79. According to the well-established guidelines [32], social support scores were defined as low (≤44) and high (> 44).

#### Fear of future violence at work scale

Fear of future WPV was measured by applying the Fear of Future Violence at Work (FFVW) scale. This is a 12-item scale applied to assess the degree to those who are afraid of experiencing violence at work during the next year [10]. Responses were rated on a 7-point Likert scale that ranged from 1 to 7 (1 = Strongly disagree to 7 = Strongly agree). The total FFVW scores ranged from 12 to 84 and higher scores indicated a higher degree of fear. To facilitate the interpretation of our findings, the total FFVW scores were classified into three groups according to the following tertiles: 12–36 (low), 37–60 (medium), 61–84 (high). The scale is valid and highly reliable, as confirmed in a previous survey [10]. To enhance its validity in our study, first, the original English version of the FFVW scale was translated into Chinese by a professional, bilingual translator. To verify its accuracy, a back-translation was performed by an independent translator. Second, we invited 15 experts to modify the items as needed, and then assessed the validity of the questionnaire content, including its suitability for Chinese culture and precision of the translation. These experts included chief physicians, clinical nurse specialists, hospital administrators, psychologists, and epidemiologists. The experts assessed the content validity of the items using the content validity index (CVI) to determine its applicability, clarification of expression, and content coverage. The scale in Chinese version was then revised as suggested by experts, and CVI values were calculated for a second assessment. Each item had a CVI value > 0.9. Third, the reliability of the scale was confirmed using a two-week test-retest (Cronbach’s alpha for the scale was 0.94) with 60 nurses, who were then excluded from the study. Cronbach’s alpha coefficient for the FFVW scores in this study (sample size 1898) was 0.97.

### Data analysis

Data were analyzed using SPSS Version 20.0 (SPSS Inc., Chicago, IL, USA). Descriptive statistics were used to analyze the scores of each item of the FFVW scale. The FFVW scores of the respondents were compared by demographic characteristics using t-test, one-way analysis of variance (ANOVA), or Kruskal-Wallis test. Variance Inflation Factor (VIF) values larger than 10 were regarded as having presence of multicollinearity [33], and VIF of all independent variables in our study were less than 5. Multiple linear regression analysis was used to examine the influencing factors of fear of future WPV. Statistical significance was set at *p* < 0.05.

### Ethical considerations

The Ethical Review Committee of the School of Nursing and Rehabilitation, Shandong University, approved the present study (Approval number: 2020-R-50). All participants provided their informed consent for enrollment before participating in the survey. Permission for use of the Fear of Future Violence at Work scale was approved from the authors via e-mail. All methods in the current study were conducted in accordance with relevant guidelines and regulations.

## Results

### Sample characteristics

Table 1 presents the demographic characteristics of the participants. Of the 1898 participants, 93.9% were female, 63.4% were aged 31–50 years, 80.1% were married, and 75.4% had at least one child. Among the participants, 88.0% received a bachelor’s degree, and 82.4% reported having a monthly income of ≥5000 Chinese Yuan (CNY). Of the participants, 39.0% were working in the surgical department and 48.4% had an intermediate professional title. Contract employees formed 78.4% of the sample. Over half of the participants (70.4%) spent less than 15 days of vacation per year, while 86.8% experienced WPV. Low levels of social support were reported by 68.9% of the participants (Table 1).

**Table 1:** Socio-demographic characteristics of respondents (*n* = 1898)

Characteristics	Number (*N* = 1898)	Percent (%)	Scores of FFVW (Mean ± SD)	F/*t*/*χ*^*2*^	*p* value
Sex				−4.823	<0.001^a^
Male	115	6.1	59.10 ± 19.22		
Female	1783	93.9	67.96 ± 16.93		
Age, years				13.281	<0.001^b^
≤ 30	621	32.7	64.85 ± 17.60		
31–50	1204	63.4	68.96 ± 16.72		
>50	73	3.8	64.11 ± 18.81		
Marital status				−4.883	<0.001^a^
Single/divorced/widowed/separated	377	19.9	63.40 ± 18.15		
Married	1521	80.1	68.43 ± 16.82		
Educational background				6.381	0.041^c^
College diploma or below	137	7.2	63.26 ± 19.67		
Bachelor	1671	88.0	67.86 ± 16.75		
Master or higher	90	4.7	65.79 ± 20.44		
Monthly income, CNY				−4.501	<0.001^a^
<5000	334	17.6	63.52 ± 17.57		
≥ 5000	1564	82.4	68.26 ± 17.02		
Number of child/children				19.450	<0.001^c^
0	466	24.6	64.75 ± 17.58		
1	907	47.8	68.59 ± 16.69		
≥ 2	525	27.7	67.80 ± 17.52		
Department				31.374	<0.001^c^
Internal medicine	538	28.3	67.18 ± 18.04		
Surgery	742	39.0	69.05 ± 16.18		
Obstetrics and gynecology	95	5.0	67.44 ± 16.52		
Pediatrics	138	7.2	69.79 ± 15.18		
Emergency department	165	8.6	66.64 ± 15.69		
Other	220	11.5	61.65 ± 19.71		
Professional status				26.626	<0.001^c^
Primary	898	47.3	65.85 ± 17.38		
Intermediate	920	48.5	69.05 ± 16.88		
Senior	80	4.2	66.53 ± 17.46		
Employment status				−0.292	0.771^a^
Regular employee	410	21.6	67.21 ± 18.83		
Contract employee	1488	78.4	67.49 ± 16.73		
Length of vacation days				13.832	0.001^c^
0	209	11.0	70.22 ± 16.22		
1–14	1129	59.5	67.76 ± 17.07		
≥ 15	560	29.5	65.71 ± 17.68		
Experience of workplace violence				−6.151	<0.001^a^
Yes	1648	86.8	68.56 ± 16.20		
No	250	13.2	59.92 ± 21.30		
Social support				2.022	0.043^a^
Low	1308	68.9	68.00 ± 16.22		
High	590	31.1	66.16 ± 19.16		

*Abbreviations*: *CNY* Chinese yuan, *USD* United States dollar, *SD* Standard Deviation

5000 CNY ≈ 718.39 USD (August 2020 exchange rate)

^a^ *p* values are derived from t-test, ^b^ *p* values were derived from One-way ANOVA, ^c^ *p* values are derived from Kruskal-Wallis Test

### Scores of fear of future violence at work

The descriptive statistics for the FFVW scores of each item provided by the participants are detailed in Table 2. We found that the average score of each item exceeded 5 points, and the average total FFVW score was 67.43, *SD* = 17.20. The item with the highest score was “If I am a victim of workplace violence, I am afraid that I will be injured” (5.81 ± 1.42, Rank 1), and the item with the lowest score was “If I encounter a potential violent individual at work, I am afraid that I will not be able to prevent a violent confrontation”(5.23 ± 1.65, Rank 12; Table 2).

**Table 2:** Score of each item and total scores of FFVW

Items	Mean ± SD	Rank
I am afraid that I will be hit, kicked, grabbed, shoved or pushed while I’m at work	5.56 ± 1.72	9
I am afraid that I will be spat on or bitten while I’m at work	5.52 ± 1.75	10
I am afraid that I will be hit with an object while I’m at work	5.41 ± 1.74	11
I am afraid that I will be threatened with any of the above examples of physical violence while I’m at work	5.64 ± 1.65	8
I am afraid that I will be threatened with a weapon while I’m at work	5.65 ± 1.72	7
I am afraid that I will be sworn at while I’m at work	5.70 ± 1.57	5
I am afraid that I will be shouted at while I’m at work	5.73 ± 1.53	4
I am afraid that someone will damage or threaten to damage my personal or workplace property while I’m at work	5.67 ± 1.62	6
I am afraid that I will be a victim of workplace violence	5.74 ± 1.60	3
If I encounter a potential violent individual at work, I am afraid that I will not be able to prevent a violent confrontation	5.23 ± 1.65	12
If I am a victim of workplace violence, I am afraid that I will be injured	5.81 ± 1.42	1
In general, I am afraid of experiencing some form of aggression, violence, or threat of aggression or violence at work	5.79 ± 1.48	2
Total scores of FFVW	67.43 ± 17.20	–

*Abbreviations*: *SD* Standard Deviation, *FFVW* fear of future violence at workplace

Rank 1–12 indicates the score of each item of FFVW from highest to lowest

### Influencing factors of fear of future workplace violence

Multiple linear regression analyses indicated that higher FFVW scores were found among those who were female (B = 7.10, SE = 1.67, *p* < 0.001), married (B = 3.50, SE = 1.60, *p* = 0.028), with monthly income ≥5000 CNY (B = 3.14, SE = 1.17, *p* = 0.007); belonged to the department of internal medicine (B = 2.90, SE = 1.35, *p* = 0.032), department of surgery (B = 5.03, SE = 1.30, *p* < 0.001), department of pediatrics (B = 5.38, SE = 1.83, *p* = 0.003), or emergency department (B = 4.50, SE = 1.74, *p* = 0.010); were contract employees (B = 2.41, SE = 1.19, *p* = 0.042); or who had experienced WPV (B = 7.02, SE = 1.15, *p* < 0.001). Lower FFVW scores were found among those who took vacation days (1–14 days: B = − 2.52, SE = 1.27, *p* = 0.047; ≥15 days: B = − 3.69, SE = 1.36, *p* = 0.007) or received a higher level of social support (B = − 2.03, SE = 0.87, *p* = 0.020; Table 3).

**Table 3:** Multivariate linear regression models for the influencing factors of FFVW analysis

Variables	Unstandardized Coefficients		Standardized Coefficients	*t*	*p* value	95% confidence interval	
	Β	SE	Beta			Lower bound	Upper bound
Sex (ref. Male)							
Female	7.10	1.67	0.098	4.261	<0.001***	3.831	10.366
Age, years (ref. ≤30)							
31–50	1.46	1.22	0.041	1.201	0.230	−0.925	3.845
>50	−1.58	2.68	−0.018	−0.592	0.554	−6.832	3.663
Marital status (ref. Single/divorced/widowed/separated)							
Married	3.50	1.59	0.081	2.197	0.028*	0.376	6.616
Education background (ref. College diploma or below)							
Bachelor	2.08	1.53	0.039	1.362	0.173	−0.915	5.070
Master or higher	0.36	2.44	0.004	0.147	0.883	−4.422	5.139
Monthly income, CNY (ref. <5000)							
≥ 5000	3.14	1.17	0.070	2.687	0.007**	0.849	5.439
Number of child/children (ref.0)							
1	−1.17	1.60	−0.034	−0.734	0.463	−4.305	1.960
≥ 2	−2.72	1.73	−0.071	−1.571	0.116	−6.111	0.674
Department (ref. Other)							
Internal medicine	2.90	1.35	0.076	2.143	0.032*	0.246	5.555
Surgery	5.03	1.30	0.143	3.869	<0.001***	2.480	7.579
Obstetrics and gynecology	3.78	2.07	0.048	1.829	0.068	−0.274	7.827
Pediatrics	5.38	1.83	0.081	2.938	0.003**	1.789	8.975
Emergency department	4.50	1.74	0.074	2.594	0.010*	1.099	7.909
Professional status (ref. Primary)							
Intermediate	1.45	1.05	0.042	1.376	0.169	−0.616	3.510
Senior	1.51	2.48	0.018	0.608	0.543	−3.354	6.371
Employment status (ref. Regular employee)							
Contract employee	2.41	1.19	0.058	2.037	0.042*	0.089	4.738
Length of vacation days (ref.0)							
1–14	−2.52	1.27	−0.072	−1.991	0.047*	−4.999	−0.038
≥ 15	−3.69	1.36	−0.098	−2.710	0.007**	−6.359	−1.020
Experience of workplace violence (ref. No)							
Yes	7.02	1.15	0.138	6.082	<0.001***	4.756	9.282
Social support (ref. Low)							
High	−2.03	0.87	−0.055	−2.337	0.020*	−3.740	−0.327

*Abbreviations*: *B* the coefficients, *SE* Standard error, *FFVW* fear of future violence at workplace

*, *p* < 0.05; **, *p* < 0.01; ***, *p* < 0.001

## Discussion

To the best of our knowledge, this is the first study of fear of future WPV and its influencing factors among nurses in Shandong, China. Our results showed that the average total score of FFVW was 67.43 ± 17.20, which indicated a high level of fear of future WPV among nurses. Fear of future WPV negatively affects the health outcomes of nurses and decreases the quality of health services for patients [34]. In addition, fear of future WPV has even led some healthcare workers to protect themselves by carrying weapons [35], which could aggravate the conflict between nurses and patients and increase the risk of work-related injury [36]. Thus, the high level of fear of WPV among nurses should be considered an important issue for hospital administrators and healthcare policymakers in China.

Previous studies have found that women report a higher level of fear of violence [37] and crime than men [38]. In our study, sex differences were found in fear of future WPV, with females reporting significantly higher scores than males. Women are usually more vulnerable and less capable of controlling their emotions and coping with the psychological damage caused by violence than men [39]. Social psychology study suggested that women with similar perceptions of the expected risk of victimization often show higher levels of fear than men [37]. In addition, a previous study revealed that male nurses have a greater ability than female nurses to think rationally and cope with emergency situations [40], which indicates that male nurses have some ability to reduce the incidence of WPV and decrease the harm from WPV as opposed to female nurses. Thus, female nurses were more likely to experience a higher level of fear of future WPV than male nurses.

In the present study, we found that married nurses experienced more fear of future WPV than those who were single/divorced/widowed/separated. This finding may be explained by the increased responsibilities of marriage. Marital status may confer specific social roles and responsibilities upon the individual and, consequently, influence the development of fear [37]. Marriage is often associated with a transition to adulthood with new roles and expectations, particularly with concerns about the welfare of other family members [37]. WPV not only had a serious negative effect on the health of nurses who were married but also might influence their spouses’ or children’s daily lives. Thus, marriage status might increase the fear of future WPV.

Our data showed that nurses with a monthly income ≥5000 CNY tended to have a higher level of fear of future WPV than those with a monthly income less than 5000 CNY. A recent study on the fear of crime found that women who were relatively wealthy had a greater fear of crime than women with moderate income. This may be explained by incidental victimization, whereby wealthy women are at risk of losing much of their property [38]. Our findings are consistent with this explanation. A possible reason for this phenomenon could be the higher opportunity cost of turnover. Opportunity cost refers to the value of the option that you reject when choosing between two possible options. Previous studies have reported that WPV has a negative effect on job satisfaction and increases turnover intention among nurses [41, 42]. Nurses who have experienced WPV often have a strong turnover intention. Nurses with a high income have a higher opportunity cost of turnover than those with a lower income. Another reason might be that nurses’ income is higher than that of the general population in China; a nurse’s income often accounts for a large proportion of the household income. According to the China Statistical Yearbook 2019 [43], 90% of people in China have a monthly income of less than 5000 CNY. WPV might result in longer work absences [44] and could reduce the income of nurses and increase the burden of living.

Our results also showed that in comparison with nurses working in other departments, those working in departments of internal medicine, surgery, pediatrics, and emergency department had higher scores of fear of future WPV. The higher prevalence of WPV in the above departments might explain this phenomenon. Similar results were reported in a previous study, in which nurses working in departments of internal medicine, surgery, pediatrics, and emergency department were more likely to experience WPV than those who were working in other departments in tertiary hospitals [20].

According to our findings, nurses who were contract employees had significantly higher scores for fear of future WPV. Regular or permanent employees can work at the hospital until their retirement; however, contract employees need to renew their contracts at regular intervals. Contract employees usually consider their socioeconomic status to be lower than that of regular employees in the same hospital. A previous study reported that the way for hospital administrators usually address medical worker-patient conflicts by reconciliation [45]. To reduce the negative impact on the hospital, hospital administrators often sacrifice nurses’ interests while handling WPV [46], such as refusal to report WPV, wage deduction, and even termination of employment contracts. This may impact the interest of the employees, especially for contract employees, which may explain the higher levels of fear of future WPV among this population.

Our findings showed that the vacation time used by participants was inversely associated with fear of future WPV. There may be two possible reasons for this phenomenon. First, compared with those who took vacations, a nurse with no vacation time would spend more time in contact with patients. A previous study found that an increase in patient contact could increase the likelihood of encountering WPV [47]. The increased probability of encountering WPV can increase the nurse’s fear of WPV. Second, nurses who take no vacation time often have heavy workloads, which lead to a higher risk of stress and anxiety [48]. Anxiety and stress might negatively impact the quality of health care which could finally affect WPV [49]. A vacation period allows nurses to spend more time with their families, to participate in leisure activities, to relax themselves, and finally to relieve their anxiety and stress levels, which could reduce the incidence of WPV. Lower expectations of encountering WPV will reduce nurses’ fear of future WPV.

The present data revealed that nurses who had experienced WPV had significantly higher scores of fear of future WPV, which indicated that the psychological stress response to WPV might have long-term effects. In addition, nurses who did not experience WPV also had an approximately high level of fear of WPV. This finding highlights that hospital administrators should take effective measures to reduce the occurrence of WPV and to establish long-term interventions to alleviate nurses’ fear of future WPV.

Our findings also showed that social support was inversely associated with fear of future WPV. Evidence from other researchers suggested that social support is an important factor in mitigating individuals’ experiences of WPV [42]. If nurses with a high level of social support encountered WPV, they might receive more encouragement and support from their family members, friends, and colleagues, so that the negative consequences of WPV would be reduced [42]. Thus, nurses with higher levels of social support tend to experience less fear of future WPV.

### Limitations

The limitations of this study are as follows: First, the survey was self-reported, so there was a risk of recall bias due to false or inaccurate responses. Second, our study was a cross-sectional study, and the causal relationship between influencing factors and fear of future WPV among nurses could not be determined. Third, the data for this study was collected from Shandong Province, China. Therefore, these results might not be generalized to nurses from other provinces. Finally, the present study only included nurses in tertiary hospitals, which may limit the generalizability of our results to other primary and secondary hospitals.

## Conclusion

Our study found a high level of fear of future WPV among nurses in tertiary hospitals in Shandong Province, China. Gender, marital status, monthly income, department, employment status, experience of WPV, vacation time, and social support were found to be influencing factors for fear of future WPV. The fear of future WPV among nurses should be considered an important issue for hospital administrators and government officials. Effective measures should be taken to reduce this fear. The factors that influenced fear of future WPV should be considered while designing and implementing interventions.

### Policy implication

This is the first study to examine fear of future WPV among nurses in Shandong Province, China. Such feeling of fear may affect nurses’ health as well as their professional performance. The present survey underlined the presence of fear of future WPV and its potential contributing factors. These findings provided insight into the creation of a safe work environment which is essential for promoting work productivity and employee’s safety. Based on the results, we suggested that hospital administrators should: (1) take effective measures, such as strengthening hospital’s security systems, further intensifying violence prevention and response systems, and improving nursing environment to control the risk factors and reduce WPV prevalence against nurses [50]. (2) provide comprehensive counseling and support services for nurses to help them reduce fear of WPV, especially for nurses who are female, married, have a monthly income of ≥5000 CNY, are working at the departments of internal medicine, surgery, pediatrics, emergency department, working as a contract employee, and have an experience of WPV, (3) enhance social support for nurses, (4) reduce nurses’ working hours and workloads, ensure nurses to spend enough vacation time, (5) develop training programs related to workplace safety for nurses, (6) improve the quality of medical services to reduce the incidence of undesirable treatment outcomes [19]. We also suggest that the government should: (1) improve the law and crack down on WPV, (2) prohibit the events of stigma toward medical staff and improve the medical staff-patient relationship.

## Data Availability

The datasets generated and/or analyzed during the current study are not publicly available due to agreements with participants who restricted data sharing but are available from the corresponding author on reasonable request.

## Abbreviations

B: The coefficients
FFVW: Fear of Future Violence at Work
GDP: Gross Domestic Product
WPV: Workplace violence
CNY: Chinese yuan
SD: Standard Deviation
SE: Standard error
SSRS: Social Support Rating Scale
USD: United States dollar
